# Validation of deep learning-based CT image reconstruction for treatment planning

**DOI:** 10.1038/s41598-023-42775-x

**Published:** 2023-09-18

**Authors:** Keisuke Yasui, Yasunori Saito, Azumi Ito, Momoka Douwaki, Shuta Ogawa, Yuri Kasugai, Hiromu Ooe, Yuya Nagake, Naoki Hayashi

**Affiliations:** 1 Division of Medical Physics, School of Medical Sciences, Fujita Health University, 1-98 Dengakugakubo, Kutsukake-Cho, Toyoake, Aichi 470-1192 Japan; 2 Department of Radiology, Fujita Health University Hospital, Toyoake, Aichi, Japan; 3 Faculty of Radiological Technology, School of Medical Sciences, Fujita Health University, Toyoake, Aichi, Japan

**Keywords:** Radiography, Radiotherapy

## Abstract

Deep learning-based CT image reconstruction (DLR) is a state-of-the-art method for obtaining CT images. This study aimed to evaluate the usefulness of DLR in radiotherapy. Data were acquired using a large-bore CT system and an electron density phantom for radiotherapy. We compared the CT values, image noise, and CT value-to-electron density conversion table of DLR and hybrid iterative reconstruction (H-IR) for various doses. Further, we evaluated three DLR reconstruction strength patterns (Mild, Standard, and Strong). The variations of CT values of DLR and H-IR were large at low doses, and the difference in average CT values was insignificant with less than 10 HU at doses of 100 mAs and above. DLR showed less change in CT values and smaller image noise relative to H-IR. The noise-reduction effect was particularly large in the low-dose region. The difference in image noise between DLR Mild and Standard/Strong was large, suggesting the usefulness of reconstruction intensities higher than Mild. DLR showed stable CT values and low image noise for various materials, even at low doses; particularly for Standard or Strong, the reduction in image noise was significant. These findings indicate the usefulness of DLR in treatment planning using large-bore CT systems.

## Introduction

Deep learning-based CT image reconstruction (DLR) is a state-of-the-art method for forming CT images^1^, and it incorporates convolutional neural networks (CNNs) into the reconstruction process^2^. It has been used to generate images at lower doses^2^ for different applications, such as MRI and CT^3^, and to reduce noise and artifacts in the reconstructed images^4–9^.

The Advanced Intelligent Clear-IQ Engine (AiCE) is a DLR algorithm developed by Canon Medical. It uses a deep convolutional neural network (DCNN) to improve image quality. The AiCE algorithm features a deep neural network that is trained using high-quality model-based IR datasets. The algorithm is trained to differentiate noise from the signal. AiCE V8 and AiCE V10 are two versions of the algorithm, with the latter reducing noise and improving spatial resolution compared with the former^10^. A comparison between DLR using AiCE and hybrid-iterative reconstruction (H-IR) showed that DLR with beam-hardening correction (AiCE Body Sharp) improves image quality in terms of noise reduction, contrast enhancement, and sharpness^11^. Many other studies have also suggested the usefulness of AiCE, which is expected to improve image quality and reduce the dose^3,10,12,13^. However, to date, the usefulness of DLR for treatment planning CT has not been investigated. For treatment planning, large-bore CT, which allows imaging of various body positions, is widely used^14^. Fixed energy and field-of-view techniques are also used to minimize changes in the Hounsfield unit (HU) value^15^. However, the quality of the image using these techniques is reduced: lower image quality leads to more variability in contouring^16,17^ and increased uncertainty in dose calculations^18,19^. Furthermore, in treatment planning CT, dose reduction should be considered following the principle of “as low as reasonably achievable”^14^. The use of DLR is expected to solve these problems and contribute to the realization of more precise delineation, improved accuracy of dose calculations, and reduced exposure doses. Therefore, investigating the characteristics of AiCE in large-bore CT for treatment planning is useful.

This study aimed to evaluate the usefulness of AiCE in radiotherapy. To this end, we compared the CT values, image noise, and CT value-to-electron density conversion table (CT-ED table) of DLR and H-IR for various doses using an electron density phantom. This study is the first to evaluate the usefulness of DLR for radiotherapy using an electron-density phantom. Improved image quality and visibility are important factors in radiotherapy.

## Results

**Figure 1:**
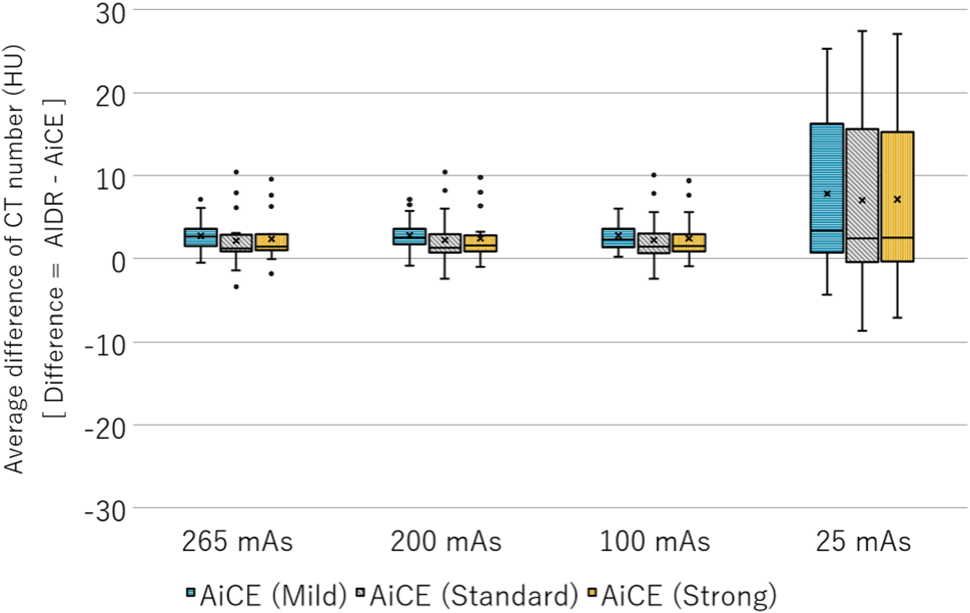
Difference in CT values between AIDR and AiCE at various doses. The difference in CT values was obtained by the difference between AIDR and AiCE; the results for the three reconstruction intensities of AiCE are shown.

**Figure 2:**
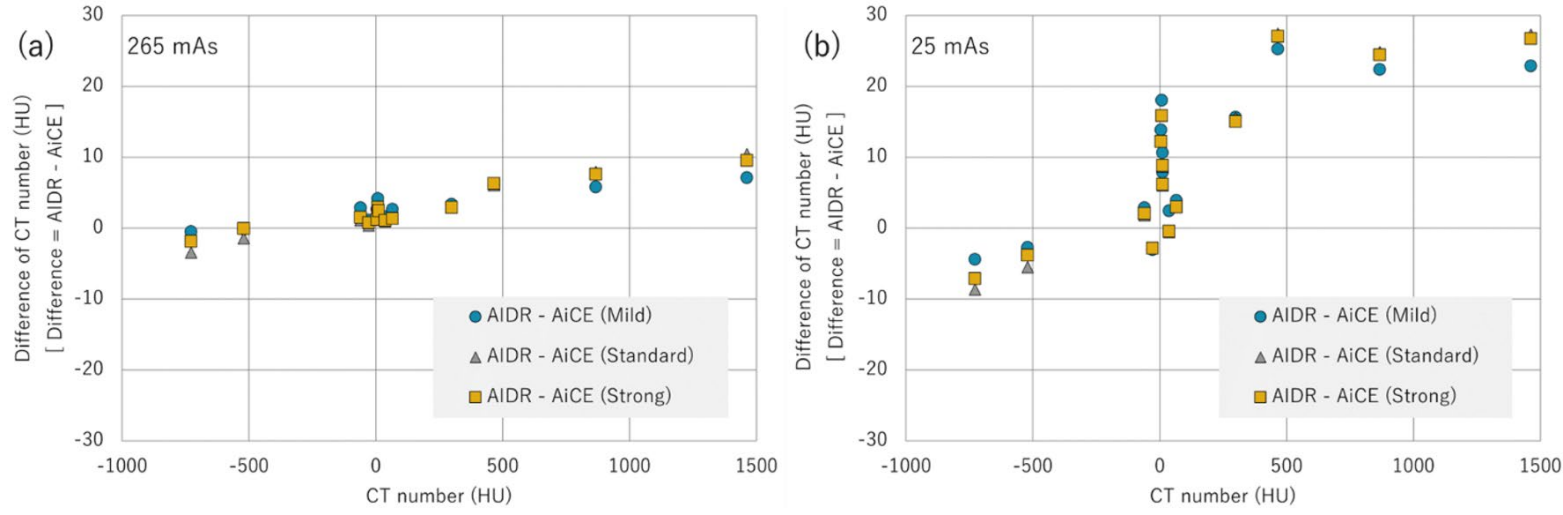
Difference in CT values between AIDR and AiCE for each material at 265 mAs (**a**) and 25 mAs (**b**). The difference in CT values was obtained by the difference between AIDR and AiCE; the results for the three reconstruction intensities of AiCE are shown.

To evaluate the effectiveness of DLR in radiotherapy, the differences between the AIDR and CT values and image variability must be examined. Figure 1 shows the differences in CT values between AIDR and AiCE at various doses; AiCE was compared at three reconstruction intensities (Mild, Standard, and Strong). The variation in CT values of all reconstruction algorithms was large at low doses, that is, 25 mAs, and the difference in average CT values was insignificant, with less than 10 HU at doses of 100 mAs and above. Regarding the change in CT values with dose, no difference was observed in AiCE reconstruction intensity. Figure 2 shows the difference in CT values between AIDR and AiCE for each material. As shown in Fig. 1, the difference was small for 265 mAs and large for 25 mAs. When a sufficient dose was ensured, the variation in CT values from the AIDR was within 10 HU, and the uncertainty of the CT values was negligible (Fig. 2a). At low doses, there were changes of 20–30 HU in high-density materials and 0–20 HU near a CT value of 0 (Fig. 2b). Regarding the differences in AiCE reconstruction intensity, Mild showed CT values closer to the AIDR than Standard/Strong for high/low-density materials, and Standard/Strong was closer to the AIDR than Mild for CT values approaching zero. The influence of the difference in reconstruction intensity was negligible as the changes were all less than 5 HU.

**Figure 3:**
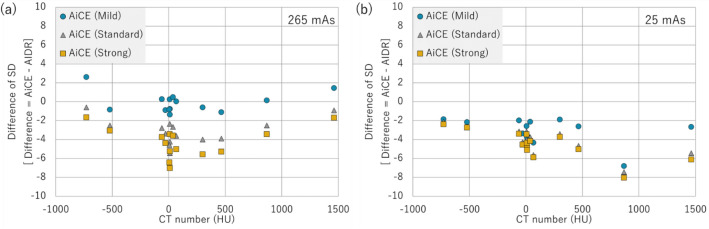
Relative SD of AiCE to AIDR for 265 mAs (**a**) and 25 mAs (**b**) for each material. Where Difference was calculated as AiCE—AIDR.

**Figure 4:**
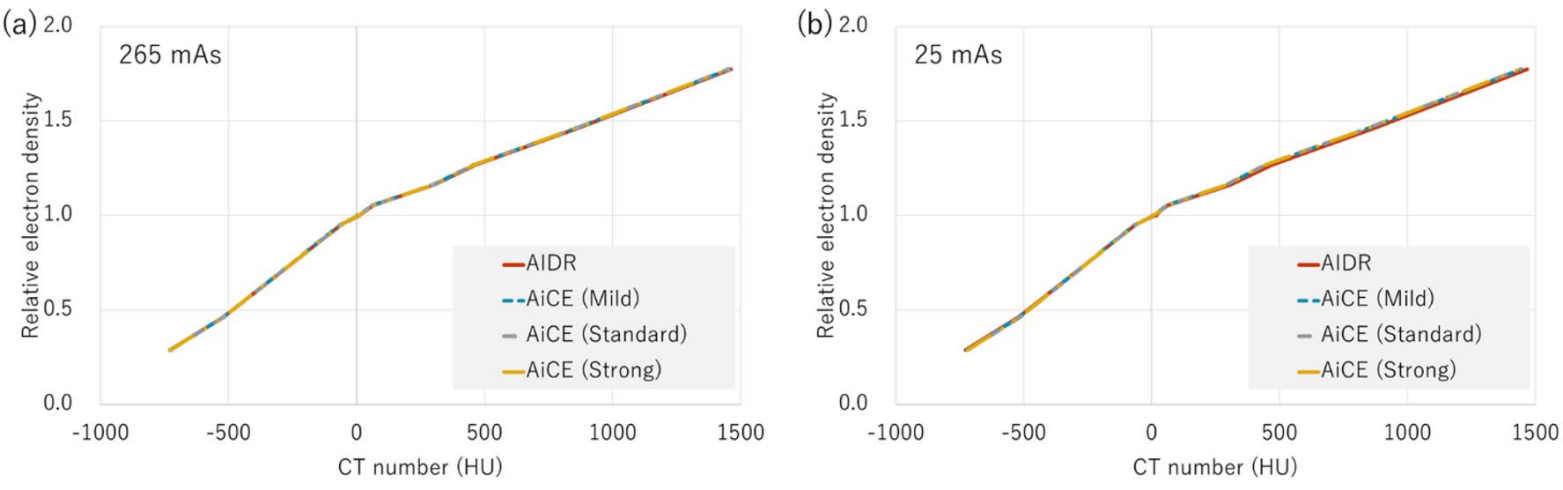
CT value-to-electron density conversion table at 265 mAs (**a**) and 25 mAs (**b**). AIDR and AiCE of the three reconstruction intensities, all four data are shown.

The variation in CT values for each material is shown in Fig. 3. SD represents the standard deviation of the CT value, with each material as the region of interest. Interestingly, from Fig. 3, it can be observed that the SD of AiCE (Strong) is the lowest for all materials and doses. AiCE (Mild) showed almost no change in SD compared with AIDR at 265 mAs. However, at 25 mAs, where the lower dose was used, AiCE had a lower SD at all reconstruction intensities than AIDR (Fig. 3b). Figure 4 shows the CT value-to-electron density conversion (CT-ED) table. Low-dose AIDR showed a slightly changed CT value (Fig. 4b), while AiCE showed no change in CT value owing to changes in reconstruction intensity and dose.

## Discussion

This study aimed to assess the usefulness of DLR (AiCE) in treatment planning using large-bore CT. The current study found that AiCE showed less change in CT values and smaller SDs than AIDR. These results suggest that AiCE can be used in radiation therapy as well as AIDR, which has been widely used, and it can be expected to play a role in recommending uncertainty in dose calculations because of the low noise in the images. The noise reduction effect was particularly large in the low-dose region (Fig. 3b), indicating that AiCE may contribute to dose reduction in CT for treatment planning. Previous studies have shown that DLR algorithms reduce noise^4–9^, and the results of this study follow similarly.

It is well known that there are variations in organ delineation, and high-quality images can reduce delineation uncertainty in treatment planning^22–25^. This is a phantom-based study, despite demonstrating little change in CT values; furthermore, combined with the benefit of improved image quality, DLR may be useful for delineation. The AiCE Mild reconstruction showed little improvement in SD, suggesting that the reconstruction intensity should be stronger than that of Mild for treatment planning CT. The reconstruction kernel of AiCE in this study only considered the Body_sharp filter, and a multifaceted study is required. Images with low variability are also important for particle therapy. In particle therapy, CT images are used for range estimation^26,27^. DLR has been shown to reduce SD, and this method can be applied to particle therapy by evaluating the accuracy of calculating the stopping power.

Dose reduction in treatment-planning CT is another important topic^28–30^. Remarkably, in this study, AiCE showed stable performance, even at low doses. The difference between AiCE and AIDR became larger for low-density materials (Fig. 2b), but this was caused by the fluctuation of the CT values of AIDR (Figs. 2 and 4b). The CT values of AiCE showed very little change with dose (Fig. 4), and at low doses, an SD reduction was observed at all reconstruction intensities (Fig. 3). The difference in SD between Mild and Standard/Strong was large, again suggesting the usefulness of reconstruction intensities that were higher than Standard.

The principal limitation of this study is that we only validated the CT-ED table at the pre-treatment planning stage and did not examine the clinical impact of differences in CT reconstruction images. Furthermore, we did not clarify the contribution of the DLR to the quality of treatment planning and dose reduction in CT treatment planning. However, the usefulness of DLR in reducing noise in clinical and low-dose CT images has been verified. This study showed a small variation in CT values and small SD in pre-treatment planning using large-bore CT; based on these results, there may be little change in dose distribution in treatment planning using patient CT data, which will contribute to the reduction of exposure dose and contouring variation owing to improved image quality. More research using patient CT data to use the DLR for treatment planning is required.

## Conclusions

This study is the first to demonstrate that the AiCE DLR algorithm is useful in radiotherapy with large-bore CT through the comparison with AIDR. Using a CT-ED tablet phantom, we found that AiCE showed stable CT values and low SD for various materials even at low doses; particularly for Standard or Strong, the reduction in SD was significant. The AiCE deep neural network trained on patient data can be applied to radiation therapy by confirming the findings obtained with the simple geometric phantom using anthropomorphic phantoms and patient datasets. Notwithstanding these limitations, this study suggests that DLR can be useful for treatment planning using large-bore CT systems.

## Materials and methods

### CT system and principles of an image reconstruction algorithm

Data were acquired using an Aquilion Exceed LB (Canon Medical). This CT has a 90 cm wide bore and wide field of view (FOV) and is available for image reconstruction using DLR and H-IR.

AiCE uses artificial intelligence nodes called “neurons”, which are networked in multiple layers to mimic human neuron connections. A deep convolutional neural network (DCNN), consisting of layers of neurons, is trained to perform complex tasks and generate CT images. The DCNN input is analyzed by several network layers, referred to as “hidden layers”. The hidden layers contain “convolutional layers”, in which the neurons act as feature selectors on small patches of data. The DCNN in AiCE has thousands of neurons and samples the feature space. For the successful performance of the DCNN, the network structure must be optimized, which affects the image quality and reconstruction speed. To achieve the best computational efficiency and improve image quality, network structure elements such as the number of network layers, the number of neurons in each layer, and convolution kernel size have been optimized in AiCE. The engine was trained using a sample dataset containing both high- and low-quality input data compared to gold standard images, allowing the system to distinguish signal from noise and reconstruct superior signal-to-noise ratios with the same radiation dose as conventional scanning techniques. To output the best optimal results, millions of image pairs were used in the training of AiCE DLR, and they were validated by thousands of phantom and patient images.

The H-IR algorithm used in this study is Adaptive Iterative Dose Reduction (AIDR). AIDR is a three-dimensional CT algorithm that reduces image noise while preserving image quality^20,21^. AIDR is a commercial H-IR algorithm that combines reconstruction and denoising in the raw data and image space domains, AIDR manipulates the projection data using noise and scanner statistical models to adjust for variations in the patient, scan parameters, and scanner itself, and adapts the filter intensity based on the relative noise levels.

### Data acquisition and reconstruction parameters

The tube voltage was 120 kV, and the tube currents were 265, 200, 100, and 25 mA. The detector configuration was 0.5 mm × 80-rows (160-slices), the rotation time was 0.5 s/rotation, and the field-of-view was LL size (500 mm). The reconstruction kernel and reconstruction strength of the AIDR were the same as those used in clinical settings (reconstruction kernel: FC13, reconstruction strength: Mild). The reconstruction kernel for AiCE was body-sharp, and the reconstruction strength was compared with three parameters: Mild, Standard, and Strong. The scan parameters are presented in Table 1.

**Table 1:** Summary of scan parameter.

X-ray tube voltage (kV)		120
X-ray tube current (mA)		265/200/100/25
Detector configuration		0.5 mm × 80-raw (160-slices)
Field-of-view		LL (500 mm)
Reconstruction parameter	AIDR	FC13, mild
	AiCE	Body_sharp, Mild/Standard/Strong

### Phantom and data analysis

**Figure 5:**
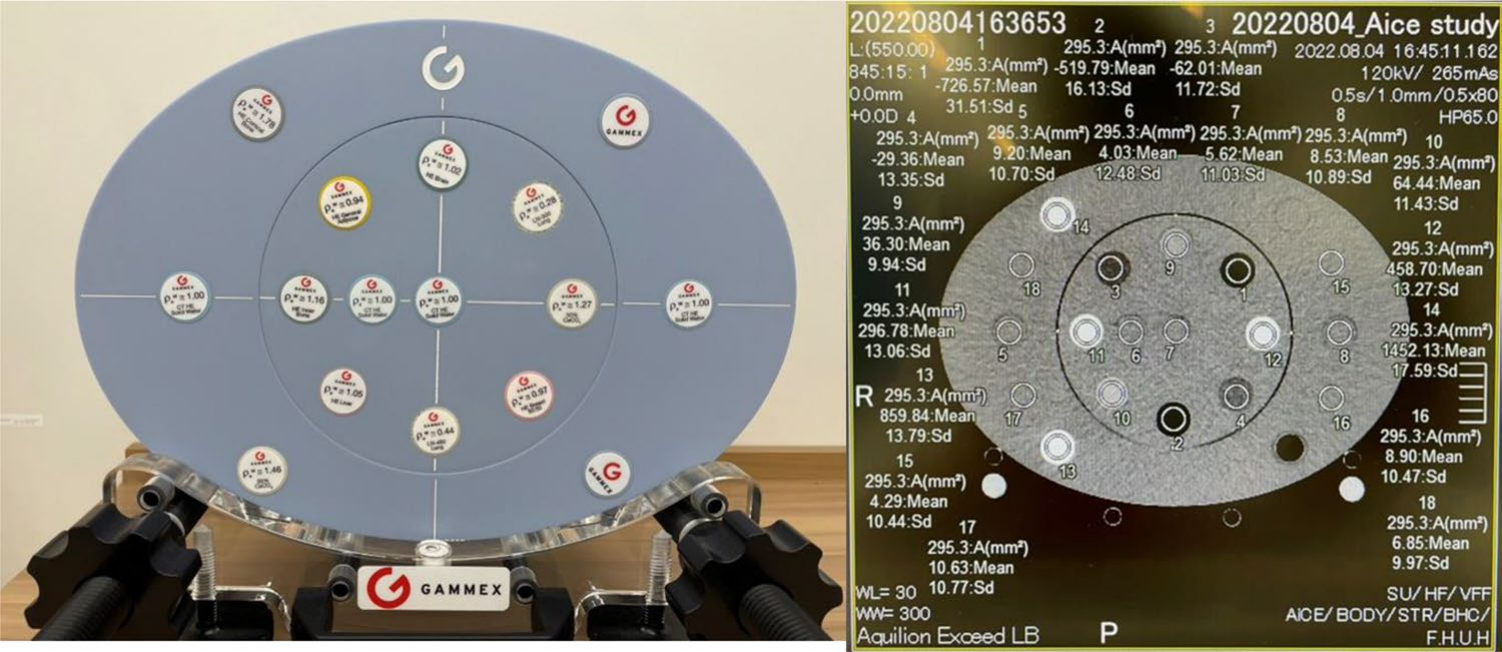
Overview (left) and analytical view (right) of CT-ED table phantom.

A large-diameter CT-ED table phantom (Advanced Electron Density Phantom; SUN NUCLEAR) was used to measure the CT values and image noise of the various materials. The rods of the advanced electron density phantom mimic water, cortical/inner bone, lung, and liver. They are highly equivalent to the medical standards for human tissue densities and can optimally convert CT values to electron density. Figure 5 shows an overview and analytical view of the phantom. In this study, 10 materials were used, ranging from the lung (electron density: 0.288 c/cm^3^) to the cortical bone (electron density: 1.774 c/cm^3^). Moreover, regions of interest (ROI) of mimicked water (electron density: 0.999 c/cm^3^) were created at the top, bottom, left, and right sides for analysis. The ROI size used in the analysis was approximately 300 mm^2^, and the ROI was set at the center of each material. CT values and variations (SD) within the ROI were analyzed. The analysis was performed using the viewer provided with the CT device. The CT value is the mean of the CT values within the specified ROI, The SD is the analysis of the variations (1σ) of CT value within the ROI.

## Data Availability

The data that support the findings of this study are available from the corresponding author upon reasonable request.
